# The prevalence of multimorbidity and associations with lifestyle factors among middle-aged Canadians: an analysis of Canadian Longitudinal Study on Aging data

**DOI:** 10.1186/s12889-019-6567-x

**Published:** 2019-02-28

**Authors:** Mohammad Nazmus Sakib, Shahin Shooshtari, Philip St. John, Verena Menec

**Affiliations:** 1 Department of Community Health Sciences, University of Manitoba, Winnipeg, Manitoba Canada; 2 Department of Internal Medicine, University of Manitoba, Winnipeg, Manitoba Canada

**Keywords:** Multimorbidity, Chronic disease, Chronic conditions, Socio-demographic factors, Lifestyle factors, CLSA

## Abstract

**Background:**

Multimorbidity can be defined as the presence of more than one chronic condition in an individual. Research on multimorbidity has predominantly focused on older adults and few studies have examined multimorbidity in middle-aged people. The objectives of this study were to: 1) examine the prevalence of multimorbidity among middle-aged Canadians; and 2) examine the association between lifestyle factors (smoking, alcohol intake, physical activity) and multimorbidity in this age group.

**Methods:**

In this analysis of the Canadian Longitudinal Study on Aging (CLSA) baseline data, we extracted data from 29,841 participants aged 45–64 years from a database of 51,338 people aged 45–85 years. Self-reported data on 27 chronic physical health conditions were used to derive different multimorbidity definitions. We estimated the prevalence of 3+ to 5+ chronic physical health conditions in different subgroups for descriptive purposes. Multivariable logistic regression analyses were performed to determine the association between socio-demographic and lifestyle factors, and multimorbidity using a 3+ multimorbidity case definition.

**Result:**

We found that 39.6% (99% CI 38.4–40.7) of participants had three or more chronic conditions with a mean number of chronic condition of 2.41 (99% CI 2.37–2.46). The prevalence of multimorbidity increased with age from 29.7% in the 45–49-year-old age group to 52% in individuals aged 60–64 years. The prevalence of 4+ and 5+ chronic conditions was 24.5 and 14.2% respectively. Analyses indicated that female sex and low income were associated with higher odds of multimorbidity, whereas daily or weekly alcohol intake were associated with lower odds of multimorbidity. Exercise was not associated with multimorbidity. Results were similar when analyses were conducted separately for women and men.

**Conclusions:**

Multimorbidity is not limited to older adults, but is a common phenomenon among middle-aged people. Longitudinal research is needed to better understand the temporal relationship between lifestyle factors and multimorbidity.

## Background

Multimorbidity, the co-existence of multiple chronic conditions in an individual [1–3], has been identified as one of the major health system concerns of the twenty-first century [4, 5]. The aging of the large baby boomer cohort, coupled with increased survival from previously fatal medical conditions, means that a growing number of adults present with multiple coexisting chronic conditions. Although multimorbidity has been considered a health problem of older adults, a substantial number of young and middle-aged people also have multimorbidity [6–8]. Despite this fact, relatively few studies have examined multimorbidity among middle-aged people.

Research based on general population samples and samples of older adults shows that multimorbidity has a major impact on individuals and the health care system. Multimorbidity negatively affects the quality of life of younger and older adults [9, 10], and significantly increases the risk of disability and mortality among older adults [11–14]. Multimorbidity also complicates the medical treatment process because multiple specialties are involved. Thus, individuals with multimorbidity are at risk of repeated hospitalization, longer hospital stays, polypharmacy, and adverse drug events [15–18]. Overall, multimorbidity increases health care utilization and costs, creating a substantial economic burden for the healthcare system [4]. Early-onset multimorbidity would be expected to further complicate this scenario as it prolongs the period of time spent in poor health as people age.

The prevalence of multimorbidity has been estimated in many studies in countries such as the United States, Australia, the Netherlands, and Canada. Its prevalence has been found to vary widely across studies - from around 20–30% when the whole population is considered to 55 to 98% in studies focusing on older adults [2, 19]. Although much of the research on multimorbidity has been conducted in Western countries [2, 19], a study conducted in China suggests that multimorbidity is common in other parts of the world as well [20]. The variation in the prevalence of multimorbidity is primarily due to differences in data sources, study populations and the number and types of chronic conditions counted to define multimorbidity [21–24]. For example, in a study using national Canadian data (the Community Health Survey [CCHS] 2011/12 data), 12.9% of Canadians aged 20 years or over had two or more chronic conditions, whereas 3.9% had three or more chronic conditions [23]. The prevalence ranged from 3.1% among individuals aged 20 to 34 to 31.3% among individuals aged 65+. In contrast, a study that recruited participants from patients of family practitioners showed a much higher prevalence of two or more chronic conditions, ranging from 68 to 93% to 99% among patients aged 18 to 44, 45 to 64, and 65+, respectively [8].

Multimorbidity is strongly associated with several sociodemographic factors. Increasing age and living in poor socioeconomic condition are established risk factors of multimorbidity [1, 23–25]. In addition, women have been found to have a higher risk of having multimorbidity than men in many (albeit not all studies), although the argument has been made that the greater longevity in women may be responsible for this higher prevalence [2, 3, 24, 26, 27]. Sex differences in health care use may also account for this finding. Similarly, some studies indicate that there is an inverse relationship between education and multimorbidity [3, 22, 28].

Researchers have also examined the relationship between lifestyle factors (smoking, alcohol intake and physical activity) and multimorbidity, but findings have been inconsistent [29]. Smoking was associated with an increased likelihood of multimorbidity among middle-aged (40–59 years old) and older participants (60+ years old) in an Australian study [30], and among men, but not women in a Canadian study [31], but decreased likelihood of multimorbidity in a South African study [32]. Being sedentary versus exercising at a low, medium, or high level was associated with increased odds of multimorbidity among older participants, but not younger participants in one study [30], whereas in another study, being physically active was associated with decreased odds of multimorbidity only among older men, but not older women [33]. Yet other research shows no relationship between physical activity and multimorbidity [31, 34]. Alcohol consumption has been found not to be associated with multimorbidity [30, 31].

In sum, although multimorbidity has been examined extensively in older adults and general population samples, few studies have focused specifically on middle-aged individuals, particularly in Canada, and examined the prevalence of multimorbidity and potential lifestyle risk factors in this age group. The objectives of this study were, therefore: 1) to examine the prevalence of multimorbidity among middle-aged Canadians; and 2) to explore the association between lifestyle factors (smoking, alcohol consumption, and physical activity) and multimorbidity in this age group.

## Methods

### Data source

This study is based on an analysis of the Canadian Longitudinal Study on Aging (CLSA) survey data [35, 36]. The CLSA is a large, national, long-term, longitudinal study which is expected to continue for at least 20 years [35, 36]. The CLSA started the recruitment process in 2010 and completed baseline data collection in 2015. The CLSA has a total sample of 51,338 Canadian women and men who were 45 to 85 years old when recruited [35, 36]. Exclusion criteria for recruitment into the CLSA were: Resident of the three territories of Canada; living on federal First Nations reserves or other First Nations settlements in the provinces; full-time members of the Canadian Armed Forces; living in long-term care institution; and cognitive impairment [35, 36].

The CLSA is comprised of a Tracking and a Comprehensive cohort. The Tracking cohort includes 21,241 participants who were randomly selected from across the 10 Canadian provinces [36]. This cohort responded to a survey only that was administered using computer-assisted telephone interview (CATI) software [37]. The Comprehensive cohort is comprised of 30,097 participants who were randomly selected from within a 25-km radius (or 50 km radius in the case of smaller cities) from one of 11 Data Collection Sites (DCS) in 7 provinces (Vancouver, Victoria, Calgary, Winnipeg, Hamilton, Ottawa, Montréal, Sherbrooke, Halifax, and St. John’s) [35]. Comprehensive cohort participants participated in computer-assisted interviews, physical assessments and provided biological samples (i.e., blood, urine) [38]. Detailed information about the sampling strategy and study design has been published elsewhere [35, 36]. Both Tracking and Comprehensive cohorts were re-interviewed with CATI software approximately 18 months after the baseline interview with a short Maintaining Contact Questionnaire [39]. Most of the variables used in the present study were derived from the baseline questionnaire; however, as physical activity was assessed only in the Maintaining Contact Questionnaire, this variable was derived from that questionnaire. Follow-up data were not yet available at the time this study was conducted.

Participants provided written informed consent to participate in the CLSA. This study was approved by the Health Research Ethics Board at the University of Manitoba.

### Study sample

As the focus of the present study was middle-aged Canadians, we included only individuals aged 45 to 64. In the total CLSA baseline sample, 12,390 and 17,541 participants from the Tracking and Comprehensive cohort, respectively were aged 45–64 years. Thus, this study included 29,841 respondents aged 45–64 years.

### Study variables

#### Multimorbidity

To determine the occurrence of chronic health conditions, respondents were asked whether a doctor ever told them that they have any one of 34 chronic conditions. From the list of chronic conditions, we included the following 27 physical health conditions: osteoarthritis, rheumatoid arthritis, other arthritis, asthma, other chronic lung diseases (emphysema, chronic bronchitis, chronic obstructive pulmonary disease, and chronic changes in lungs due to smoking), hypertension, diabetes, chronic heart disease (congestive heart failure), ischemic heart disease, peripheral vascular disease, cerebrovascular disease, Parkinson’s disease, multiple sclerosis, epilepsy, migraine, intestinal or stomach ulcers, chronic bowel disorders (Crohn’s Disease, ulcerative colitis, or Irritable Bowel Syndrome), cataract, glaucoma, macular degeneration, cancer, allergy, osteoporosis, back problem, hypothyroidism or myxedema, hyperthyroidism or Graves’ disease, and kidney disease or kidney failure.

The types of chronic conditions included in multimorbidity studies varies widely, as does the number of chronic conditions, which have ranged from 11 to 130 [19]. We included the 27 chronic physical health conditions listed above as they either fit with the definition of a chronic condition or have been included in previous multimorbidity studies [2, 19, 24]. In some instances, we collapsed related chronic conditions to bring them under the same headings. For example, angina and myocardial infarction were collapsed into ischaemic heart diseases; transient ischemic attack and cerebrovascular accident were collapsed as cerebrovascular diseases.

Multimorbidity was defined in this study as the presence of three or more chronic physical health conditions in an individual, consistent with previous research [23, 24]. We also estimated the prevalence of 4+ and 5+ chronic physical health conditions for descriptive purposes to better understand the chronic disease burden among middle-aged people.

#### Socio-demographic and lifestyle variables

Socio-demographic and lifestyle factors related data were based on self-report, and included respondents’ age, sex, educational level, annual household income, smoking habit, alcohol intake, and physical activity.

#### Age

Participants’ age was calculated from their date of birth. It was converted into an ordinal variable with the following age groups: 45–49, 50–54, 55–59 and 60–64.

#### Sex

Participants were asked about their sex, with men coded as 1 and women coded as 2.

#### Income

The socioeconomic status of the participants was assessed with household income. Participants were asked: “What is your best estimate of the total household income received by all household members, from all sources, before taxes and deductions, in the past 12 months?” [37]. This variable was categorized as follows: 1 = Less than $20,000; 2 = $20,000 or more, but less than $50,000; 3 = $50,000 or more, but less than $100,000; 4 = $100,000 or more, but less than $150,000; and 5 = $150,000 or more. There were some missing values (*n* = 1393) which were coded as 6 = “No Response”.

#### Education

The level of education was obtained from two variables. After first responding to whether they had graduated from high school, participants were asked: “Have you received any other education that could be counted towards a degree, certificate, or diploma from an educational institution?” [37]. Respondents who said “No” were considered as having an education which was “high school or less”. Participants who said “Yes” were further asked the following question to know the level of education achieved: “What is the highest degree, certificate, or diploma you have obtained?”: no post-secondary degree, certificate, or diploma; trade certificate or diploma from a vocational school or apprenticeship training, non-university certificate or diploma from a community college, school of nursing, etc.; university certificate below bachelor’s level; Bachelor’s degree; and university degree or certificate above bachelor’s degree [37]. An ordinal variable was derived from these questions with the following categories: high school or less; non-college or non-university degree; and college or university degree.

#### Smoking

Participants were asked: “At the present time, do you smoke cigarettes daily, occasionally or not at all?” with the following response options: Daily (at least one cigarette every day for the past 30 days); Occasionally (at least one cigarette in the past 30 days, but not every day); Not at all (you did not smoke at all in the past 30 days); Don’t know/No answer, and Refused [37]. This variable was recoded as 0 = non-smoker (not at all), 1 = smoker (occasionally or daily) and 2 = no response.

#### Alcohol

Participants were asked: “About how often during the past 12 months did you drink alcohol?” with the following response options: Almost every day (including 6 times a week); 4–5 times a week; 2–3 times a week; Once a week, 2–3 times a month; About once a month; Less than once a month; Never; Don’t know/No answer, and Refused [37]. This variable was recoded as follows: 0 = No drinker, 1 = Occasional drinker (about once a month or less than once a month), 2 = Weekly drinker (once a week or 2–3 times a month), 3 = Daily drinker (almost every day or 4–5 times a week), and 4 = no response.

#### Physical activity

Participants were asked: “Over the past 7 days, how often did you engage in moderate sports or recreational activities such as ballroom dancing, hunting, skating, golf without a cart, softball or other similar activities?” with the following response options: Never; Seldom (1 to 2 days); Sometimes (3 to 4 days); Often (5 to 7 days); Don’t know/No answer, and Refused [39]. This variable was recoded as follows: 0 = never, 1 = seldom, 2 = sometimes or often, and 3 = no response.

#### Covariates

CLSA participants’ area of residence (urban/rural) and province of residence, depressive symptoms, and functional status were included as covariates in the multivariable analyses. Area of residence and province of residence we controlled for to offset the effect of any regional variation and the study design. Area of residence was defined as rural or urban. This variable was provided in the dataset as: Rural; Urban core; Urban fringe; Urban population centre outside a Census Metropolitan Area and Census Agglomeration; Secondary core; and Postal code link to dissemination area. The variable was recoded as 0 = rural and 1 = urban, with the latter including all non-rural categories. All ten Canadian provinces were included in the analyses, as participants were recruited from all provinces. The inclusion of the ten provinces as a covariate in analyses is recommended by CLSA, given the study’s sampling design [40].

Given that our definition of multimorbidity focused on physical health problems, we included depressive symptoms as a covariate to control for the possible confounding effect of mental health problems in the relationship between socio-demographic and lifestyle factors and multimorbidity. Depressive symptoms were measured using the Center for Epidemiologic Studies Short Depression Scale (CES-D10) [41]. A depressive symptom score was derived based on 10 questions regarding feelings of depression, loneliness, hopefulness for the future, and restless sleep where each question has four response options (all of the time, occasionally, some of the time, rarely or never) [41]. The total score can range from 0 to 30. We recoded this scale as no depressive symptoms (score 0–9) and depressive symptoms (score 10 or more).

Functional status was controlled for as it is related to both socio-demographic characteristics and multimorbidity. Controlling for functional status therefore allows examining the unique association between socio-demographic and lifestyle factors and multimorbidity. A modified version of the Older Americans Resources and Services (OARS) Multidimensional Assessment Questionnaire was used to assess functional limitations [42]. Functional status was categorized as no, mild, moderate, severe and total functional limitations. Considering the comparatively younger cohort of our study, we collapsed the mild, moderate, severe and total functional limitation categories to create a no functional limitations and some limitations variable.

### Statistical analyses

The prevalence of multimorbidity for the middle-aged population, stratified by all the variables was first estimated. In addition, the mean number of chronic conditions was calculated for each subgroup. We were also interested to measure the most common chronic conditions occurring in middle-aged people. We, therefore, estimated the prevalence of all 27 chronic conditions for the total sample, as well as stratified by sex.

To identify the factors independently associated with multimorbidity, we performed multivariable logistic regression analysis where we included the socio-demographic and lifestyle variables and controlled for covariates (area of residence, provinces, depression, and functional limitations). Separate analyses were also conducted for men and women. For these analyses, we used 3+ chronic physical health conditions as the case definition. Sensitivity analyses were also conducted using the 4+ and 5+ multimorbidity definitions.

To make the estimates generalizable to the Canadian population and address the complexity of the CLSA survey design, we used trimmed weights in the descriptive analyses and analytic weights in regression analyses, as recommended by CLSA [40]. Weights are calculated by CLSA and are provided in the dataset that is released to researchers [40]. Statistical analyses and data management were performed using SAS software package Version 9.4 (SAS Institute Inc., Cary, North Carolina, USA).

## Results

### Sample description

Men (49.4%) and women (50.6%) were equally represented in the sample with an average age of 54.6 years (99% confidence interval [CI] 54.44–54.69) (Table 1). Most of the respondents had either a college or university degree (63.9%) (Table 1). A substantial proportion of respondents (84.3, 99% CI 83.47–85.21) reported at least one of the chronic physical health conditions included in this study, with an overall average of 2.41 (99% CI 2.37–2.46) chronic conditions per person (Table 1).

**Table 1:** Sample description

Variables	Percentage (%)	Mean chronic conditions (99% CI)
Total sample	100	2.41 (2.37, 2.46)
Sex		
Male	49.40	2.14 (2.08, 2.20)
Female	50.60	2.68 (2.61, 2.75)
Age group		
45–49	20.50	1.95 (1.85, 2.05)
50–54	34.38	2.14 (2.06, 2.22)
55–59	21.23	2.64 (2.54, 2.74)
60–64	23.89	3.00 (2.91, 3.10)
Education		
Either college or university degree	63.90	2.27 (2.21, 2.32)
No college or university degree	19.23	2.59 (2.48, 2.71)
High school or less	16.87	2.77 (2.63, 2.90)
Household income		
< $20,000	4.00	3.83 (3.54, 4.13)
$20,000 to < $50,00	15.58	2.92 (2.78, 3.05)
$50,000 to < $100,000	33.73	2.39 (2.31, 2.47)
$100,000 to < $150,000	22.64	2.18 (2.09, 2.27)
< $150,000 or more	20.10	1.96 (1.87, 2.06)
No response	3.95	2.86 (2.60, 3.12)
Smoking status		
Non-smoker	55.22	2.46 (2.39, 2.52)
Smoker	12.65	2.71 (2.55, 2.86)
No response	31.13	2.23 (2.17, 2.29)
Alcohol intake		
Never	10.11	3.13 (2.96, 3.30)
Occasional	20.50	2.78 (2.66, 2.90)
Weekly	23.40	2.25 (2.16, 2.35)
Daily	43.95	2.16 (2.10, 2.23)
No response	2.04	2.47 (2.17, 2.77)
Moderate physical activity		
Never	78.89	2.44 (2.39, 2.49)
Seldom	8.31	2.12 (1.97, 2.27)
Sometimes or often	4.80	2.11 (1.93, 2.30)
No response	8.01	2.66 (2.47, 2.84)
Area of residence		
Urban	78.69	2.41 (2.36, 2.47)
Rural	21.31	2.42 (2.30, 2.53)
Province of residence		
Alberta	9.86	2.37 (2.26, 2.48)
British Columbia	13.85	2.47 (2.39, 2.55)
Manitoba	3.34	2.31 (2.20, 2.42)
New Brunswick	2.40	2.68 (2.46, 2.90)
Newfoundland and Labrador	1.74	2.58 (2.42, 2.74)
Nova Scotia	3.03	2.47 (2.32, 2.63)
Ontario	38.02	2.47 (2.37, 2.57)
Prince Edward Island	0.44	2.41 (2.20, 2.61)
Quebec	24.57	2.29 (2.20, 2.38)
Saskatchewan	2.76	2.39 (2.19, 2.58)
Depression		
No depression	83.35	2.24 (2.19, 2.29)
Depressive symptoms	16.65	3.25 (3.10, 3.39)
Functional limitations		
No limitations	93.50	2.25 (2.21, 2.30)
Some limitations	6.50	4.60 (4.35, 4.85)

Note: Weighted percentages and means are shown

### Prevalence of multimorbidity

**Fig. 1:**
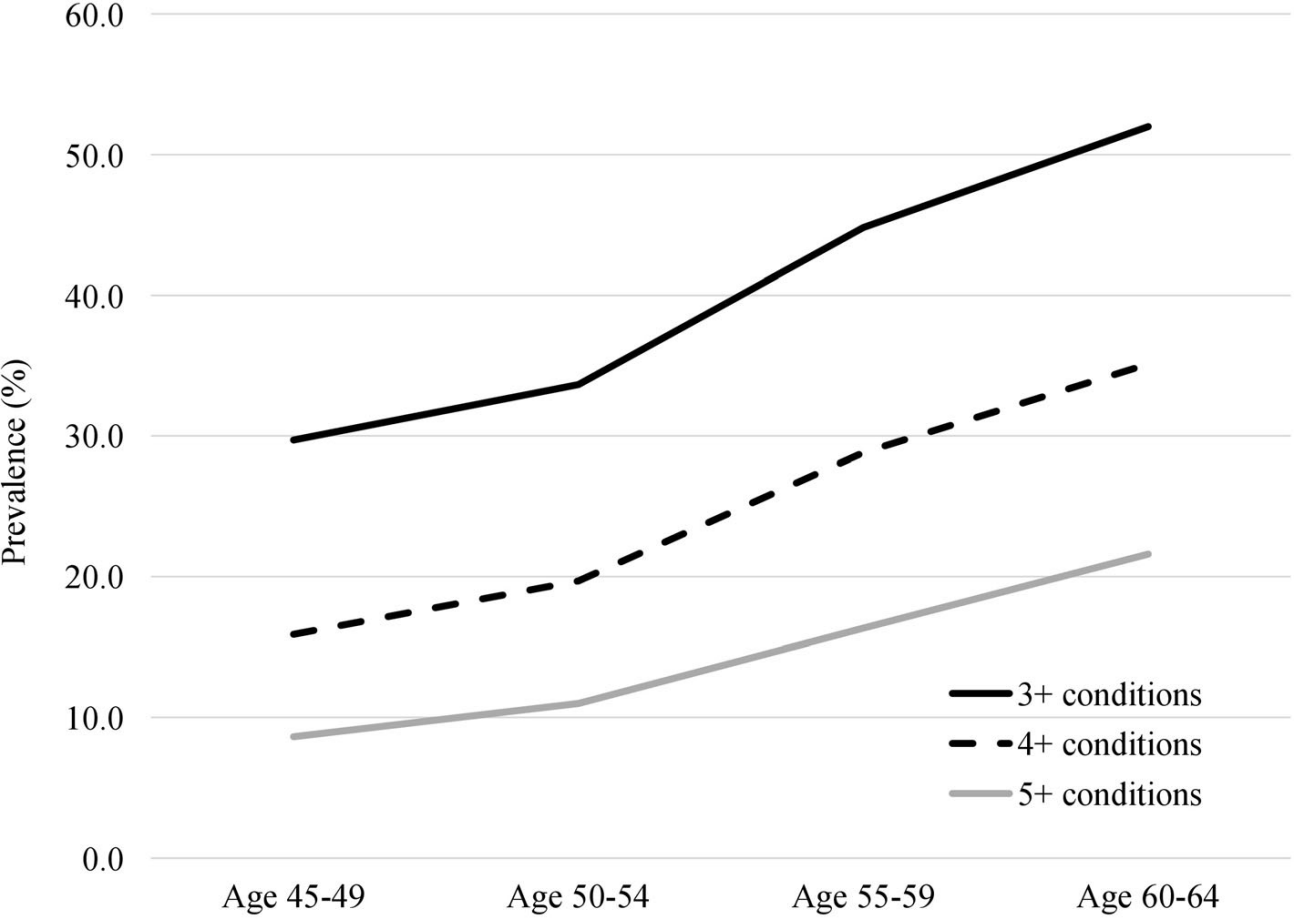
The prevalence of 3+ to 5+ chronic conditions increases with age

The crude prevalence of multimorbidity, defined as three or more chronic conditions in an individual, for the full study sample was 39.6% (99% CI 38.44–40.74). The prevalence of 4+ and 5+ chronic conditions was 24.5% (99% CI 23.52–25.54) and 14.2% (99% CI 13.37–14.96) respectively (Table 2). The prevalence of multimorbidity increased steadily with age, being 29.7% in participants aged 45–49 years, 33.7% in those aged 50–54 years, 44.8% in those aged 55–59 years and 52% in those aged 60–64 years (Table 2). This pattern was evident for all multimorbidity definitions - from 3+ to 5+ chronic conditions (Fig. 1). The prevalence of multimorbidity was higher in women (45, 99% CI 43.32–46.62) than in men (34.1, 99% CI 32.50–35.67). The prevalence of multimorbidity was slightly lower in individuals reporting higher education (Table 2). We found an inverse relationship between multimorbidity and income. The prevalence of multimorbidity decreased steadily as income increased (Table 2). The prevalence of multimorbidity was slightly higher among participants who never smoked compared to smokers, those with a daily alcohol consumption, and those who never exercised. Similar patterns across sociodemographic and lifestyle factors emerged irrespective of the multimorbidity definition used (3+ to 5+) (Table 2).

**Table 2:** Prevalence of multimorbidity across socio-demographic characteristics and lifestyle variables

Variables	Prevalence of multimorbidity (weighted %, 99 CI)		
	3+ chronic conditions	4+ chronic conditions	5+ chronic conditions
Total sample	39.59 (38.44, 40.74)	24.53 (23.52, 25.54)	14.17 (13.37, 14.96)
Sex			
Male	34.09 (32.50, 35.67)	19.65 (18.33, 20.97)	10.65 (9.66, 11.65)
Female	44.97 (43.32, 46.62)	29.30 (27.79, 30.80)	17.61 (16.36, 18.87)
Age group			
45–49	29.70 (27.10, 32.30)	15.89 (13.75, 18.02)	8.62 (6.97, 10.27)
50–54	33.65 (31.64, 35.66)	19.69 (17.98, 21.40)	10.99 (9.66, 12.32)
55–59	44.81 (42.40, 47.22)	28.81 (26.62, 31.00)	16.33 (14.57, 18.10)
60–64	52.00 (49.84, 54.16)	35.11 (33.04, 37.18)	21.60 (19.82, 23.38)
Education			
College/university degree	37.08 (35.67, 38.50)	21.72 (20.52, 22.91)	11.94 (11.01, 12.87)
Non-college/university degree	41.94 (39.33, 44.55)	27.73 (25.37, 30.09)	17.02 (15.07, 18.97)
High school or less	46.43 (43.48, 49.37)	31.54 (28.76, 34.32)	19.40 (17.06, 21.73)
Household income			
< $20,000	60.31 (55.18, 65.43)	46.58 (41.38, 51.79)	36.50 (31.47, 41.53)
$20,000 to < $50,00	49.54 (46.67, 52.40)	33.86 (31.09, 36.64)	21.08 (18.68, 23.47)
$50,000 to < $100,000	39.96 (37.97, 41.95)	24.64 (22.91, 26.38)	13.60 (12.22, 14.97)
$100,000 to < $150,000	35.03 (32.61, 37.44)	19.92 (17.88, 21.96)	10.49 (8.96, 12.03)
< $150,000 or more	30.49 (28.00, 32.99)	16.05 (14.08, 18.03)	8.03 (6.63, 9.44)
No response	48.71 (43.40, 54.02)	33.97 (28.88, 39.07)	21.61 (16.98, 26.24)
Smoking status			
Non-smoker	40.46 (38.91, 42.00)	25.14 (23.78, 26.51)	14.57 (13.48, 15.67)
Smoker	43.98 (40.69, 47.27)	29.40 (26.40, 32.39)	18.86 (16.29, 21.43)
No response	36.38 (34.85, 37.92)	21.56 (20.25, 22.88)	11.64 (10.65, 12.64)
Alcohol intake			
Never	50.57 (46.99, 54.15)	35.95 (32.53, 39.36)	24.96 (21.86, 28.06)
Occasional	46.48 (43.88, 49.07)	31.11 (28.73, 33.49)	18.73 (16.74, 20.72)
Weekly	36.81 (33.53, 36.96)	21.86 (19.84, 23.89)	12.28 (10.68, 13.89)
Daily	35.24 (33.53, 36.96)	20.18 (18.73, 21.63)	10.41 (9.33, 11.49)
No response	41.67 (34.12, 49.23)	26.18 (19.77, 32.60)	17.77 (12.20, 23.34)
Moderate physical activity			
Never	40.08 (38.79, 41.38)	24.87 (23.73, 26.00)	14.33 (13.43, 15.24)
Seldom	33.57 (29.73, 37.41)	18.72 (15.50, 21.94)	10.28 (7.80, 12.76)
Sometimes or often	35.22 (30.33, 40.12)	19.39 (15.39, 23.40)	11.36 (8.10, 14.62)
No response	43.67 (39.32, 48.01)	30.34 (26.27, 34.41)	18.35 (14.90, 21.80)
Area of residence			
Urban	39.57 (38.32, 40.83)	24.26 (23.16, 25.36)	14.10 (13.22, 14.98)
Rural	39.67 (36.92, 42.43)	25.52 (23.07, 27.96)	14.45 (12.51, 16.40)
Province of residence^a^			
Alberta	39.22 (36.47, 41.98)	24.20 (21.77, 26.62)	14.31 (12.32, 16.30)
British Columbia	41.28 (39.25, 43.32)	25.28 (23.51, 27.06)	15.10 (13.66, 16.54)
Manitoba	37.43 (34.71, 40.16)	22.74 (20.38, 25.09)	13.03 (11.17, 14.89)
New Brunswick	43.30 (38.64, 47.96)	28.70 (24.47, 32.92)	18.66 (15.01, 22.31)
Newfoundland and Labrador	44.81 (40.84, 48.77)	27.18 (23.67, 30.70)	15.95 (13.07, 18.83)
Nova Scotia	41.25 (37.58, 44.92)	25.01 (21.82, 28.20)	14.43 (11.84, 17.01)
Ontario	40.53 (38.13, 42.94)	25.84 (23.71, 27.97)	14.64 (12.94, 16.34)
Prince Edward Island	40.63 (35.42, 45.84)	24.73 (20.18, 29.29)	13.94 (10.28, 17.60)
Quebec	36.73 (34.58, 38.88)	21.94 (20.09, 23.80)	12.49 (11.03, 13.94)
Saskatchewan	39.19 (34.66, 43.71)	23.27 (19.41, 27.13)	13.79 (10.63, 16.94)
Depression			
No depression	36.33 (35.08, 37.58)	21.49 (20.42, 22.56)	11.75 (10.92, 12.58)
Depressive symptoms	55.05 (52.17, 57.94)	38.76 (35.97, 41.55)	25.77 (23.28, 28.27)
Functional limitations			
No limitations	36.90 (35.73, 38.08)	21.81 (20.81, 22.82)	11.87 (11.09, 12.65)
Some limitations	75.79 (72.04, 79.53)	61.03 (56.65, 65.41)	45.20 (40.69, 49.72)

Note: Weighted percentages are shown. ^a^Provinces are listed in alphabetic order

We also estimated the mean number of chronic conditions in different subgroups of the sample. For example, consistent with the prevalence estimates, the mean number of chronic conditions increased with age and was higher in women (2.7, 99% CI 2.61–2.75) than men (2.1, 99% CI 2.08–2.20) (Table 1).

### Common chronic conditions

Among the 27 chronic conditions included in the study, allergy was the most prevalent chronic condition (40.2, 99% CI 39.07–41.40) (Table 3). Hypertension 26.2% (99% CI 25.19–27.23) and back problem 24.7% (99% CI 23.71–25.73) were the second and third most common conditions (Table 3). Some other common chronic conditions were osteoarthritis (18.9%), diabetes (12.9%), migraine (15.6%), asthma (12.5%) and other arthritis (11.4%) (Table 3). When the prevalence of chronic conditions was stratified by sex, men were found to have a higher prevalence of hypertension (28.5% vs. 24%), back problems (26.2% vs. 23.3%), diabetes (13.7% vs. 12.2%), stomach/intestinal ulcer (6.4% vs. 6.1%), heart disease (CHF) (7% vs. 4%), ischemic heart disease (5.8% vs.2.7%), cerebrovascular disease (2.4% vs. 1.8%), kidney disease (2.1% vs. 1.5%), and Parkinson disease (0.3% vs. 0.1%) (Table 3). For the other conditions, women had a higher prevalence than men (Table 3).

**Table 3:** Prevalence of specific chronic conditions

Chronic condition	Prevalence (weighted %, 99 CI)		
	Total sample	Men	Women
Allergy	40.23 (39.07, 41.40)	34.38 (32.77, 35.98)	54.06 (52.40, 55.72)
Hypertension	26.21 (25.19, 27.23)	28.47 (26.99, 29.96)	24.00 (22.59, 25.40)
Back problem	24.72 (23.71, 25.73)	26.17 (24.71, 27.63)	23.30 (21.91, 24.70)
Osteoarthritis	18.88 (17.96, 19.81)	15.07 (13.85, 16.28)	22.60 (21.22, 23.98)
Migraine	15.59 (14.71, 16.46)	8.92 (7.92, 9.91)	22.09 (20.69, 23.50)
Diabetes	12.90 (12.11, 13.68)	13.65 (12.53, 14.76)	12.17 (11.07, 13.26)
Asthma	12.48 (11.71, 13.26)	11.07 (10.01, 12.13)	13.86 (12.73, 14.99)
Other arthritis	11.43 (10.65, 12.21)	9.70 (8.69, 10.72)	13.12 (11.94, 14.30)
Cancer	9.20 (8.52, 9.88)	7.49 (6.62, 8.35)	10.87 (9.83. 11.92)
Chronic bowel disease (IBS, IBD)	7.96 (7.32, 8.59)	5.33 (4.58, 6.08)	10.51 (9.51, 11.52)
Cataract	7.88 (7.28, 8.48)	7.00 (6.19, 7.82)	8.74 (7.87, 9.62)
Hypothyroidism	7.07 (6.43, 7.71)	3.19 (2.54, 3.84)	10.86 (9.79, 11.94)
Stomach/intestinal ulcer	6.24 (5.67, 6.81)	6.40 (5.56, 7.24)	6.07 (5.30, 6.85)
Heart disease/CHF	5.46 (4.95, 5.97)	6.95 (6.14, 7.75)	4.01 (3.38, 4.64)
Osteoporosis	5.24 (4.72, 5.75)	1.77 (1.34, 2.21)	8.62 (7.70, 9.54)
Peripheral vascular disease	4.67 (4.18, 5.16)	4.29 (3.62, 4.96)	5.04 (4.32, 5.75)
Emphysema/chronic bronchitis/COPD	4.46 (4.00, 4.93)	4.17 (3.54, 4.80)	4.74 (4.07, 5.42)
Ischemic heart disease	4.22 (3.75, 4.70)	5.82 (5.05, 6.59)	2.66 (2.11, 3.22)
Hyperthyroidism	4.04 (3.69, 4.40)	2.02 (1.62, 2.42)	6.03 (5.44, 6.61)
Rheumatoid arthritis	3.81 (3.34, 4.28)	3.42 (2.79, 4.05)	4.19 (3.49, 4.90)
Glaucoma	2.11 (1.79, 2.43)	1.93 (1.50, 2.36)	2.28 (1.81, 2.75)
Cerebrovascular disease	2.10 (1.77, 2.44)	2.41 (1.88, 2.94)	1.80 (1.39, 2.22)
Kidney disease	1.82 (1.53, 2.11)	2.12 (1.65, 2.59)	1.53 (1.18, 1.87)
Macular degeneration	1.51 (1.23, 1.79)	1.40 (1.03, 1.76)	1.61 (1.19, 2.03)
Epilepsy	0.97 (0.74, 1.19)	0.89 (0.59, 1.18)	1.05 (0.71, 1.39)
Multiple sclerosis	0.66 (0.51, 0.82)	0.41 (0.24, 0.57)	0.92 (0.65, 1.18)
Parkinson disease	0.19 (0.09, 0.30)	0.28 (0.08, 0.47)	0.11 (0.02, 0.19)

Note: Weighted percentages are shown. *IBS* = Irritable Bowel Syndrome, *IBD* = Inflammatory Bowel Disease, *CHF* = Congestive Heart Failure, *COPD* = Chronic Obstructive Pulmonary Disease

### Regression results

Multivariable logistic models using 3+ chronic physical health conditions versus < 3 conditions as the outcome variable showed that multimorbidity was significantly associated with several socio-demographic factors (Table 4). Female sex and lower income were associated with higher odds of 3+ chronic conditions, whereas weekly and daily alcohol intake was associated with lower odds of having multimorbidity. Education, smoking, and physical activity were not significantly associated with multimorbidity. Significant associations also emerged for the covariates, with depressive symptoms and functional limitations associated with higher odds of multimorbidity. Results were similar for women and men (Table 4). Similar results were also obtained in the sensitivity analyses in which we used 4+ and 5+ chronic conditions as outcome variables.

**Table 4:** Multivariable analyses of the association between sociodemographic and lifestyle characteristics and multimorbidity

Variables	3+ vs. < 3 Chronic Physical Health Conditions Adjusted Odds Ratios (99% CI)		
	Total sample	Men	Women
Sex			
Male	1	–	–
Female	1.48 (1.38,1.59)	–	–
Age group			
45–49	1	1	1
50–54	1.29 (1.16,1.44)	1.33 (1.13,1.58)	1.27 (1.10,1.47)
55–59	1.98 (1.78,2.21)	2.06 (1.74,2.43)	1.93 (1.66,2.23)
60–64	2.79 (2.50,3.11)	2.76 (2.34,3.24)	2.84 (2.44,3.29)
Education			
College/university degree	1	1	1
Non-college/university degree	1.07 (0.98,1.18)	1.14 (1.00,1.30)	1.00 (0.88,1.14)
High school or less	1.04 (0.94,1.15)	1.08 (0.93,1.27)	0.99 (0.86,1.14))
Household income			
≥$150,000	1	1	1
$100,000 to < $150,000	1.03 (0.93,1.15)	0.97 (0.83,1.13)	1.10 (0.94,1.28)
$50,000 to < $100,000	1.12 (1.01,1.25)	1.11 (0.96,1.28)	1.14 (0.98,1.22)
$20,000 to < $50,00	1.26 (1.11,1.43)	1.25 (1.03,1.51)	1.27 (1.07,1.51)
< $20,000	1.61 (1.32,1.96)	1.54 (1.14,2.07)	1.69 (1.29,2.21)
No response	1.20 (1.10,1.45)	1.20 (0.90,1.61)	1.21 (0.95,1.54)
Smoking status			
Non-smoker	1	1	1
Smoker	0.97 (0.87,1.09)	0.94 (0.80,1.11)	1.00 (0.86,1.17)
No response	0.84 (0.78,0.91)	0.76 (0.67,0.85)	0.92 (0.83,1.02)
Alcohol intake			
Never	1	1	1
Occasional	0.99 (0.87,1.13)	1.01 (0.83,1.23)	0.96 (0.81,1.15)
Weekly	0.75 (0.66,0.86)	0.83 (0.68,1.00)	0.70 (0.58,0.83)
Daily	0.69 (0.61,0.77)	0.71 (0.60,0.84)	0.67 (0.56,0.80)
No response	0.78 (0.61,1.01)	0.92 (0.61,1.39)	0.69 (0.50,0.95)
Moderate physical activity			
Sometimes or often	1	1	1
Seldom	0.98 (0.80,1.20)	0.91 (0.68,1.22)	1.07 (0.81,1.41)
Never	1.12 (0.94,1.32)	1.02 (0.80,1.29)	1.23 (0.98,1.55)
No response	1.08 (0.87,1.33)	0.95 (0.70,1.28)	1.23 (0.92,1.64)
Area of residence			
Urban	1	1	1
Rural	0.94 (0.85,1.04)	0.90 (0.77,1.04)	0.97 (0.85,1.11)
Province of residence^a^			
Quebec	1	1	1
Alberta	1.16 (1.01,1.34)	1.07 (0.86,1.33)	1.25 (1.03,1.51)
British Columbia	1.28 (1.13,1.44)	1.28 (1.07,1.52)	1.28 (1.09,1.50)
Manitoba	1.07 (0.93,1.24)	1.13 (0.91,1.40)	1.03 (0.85,1.24)
New Brunswick	1.21 (0.97,1.50)	0.98 (0.71,1.37)	1.44 (1.07,1.94)
Newfoundland and Labrador	1.35 (1.15,1.57)	1.23 (098,1.54)	1.44 (1.17,1.77)
Nova Scotia	1.20 (1.04,1.38)	1.30 (1.06,1.60)	1.11 (0.91,1.34)
Ontario	1.41 (1.26,1.58)	1.44 (1.22,1.70)	1.38 (1.19,1.61)
Prince Edward Island	1.13 (0.88,1.44)	1.02 (0.71,1.47)	1.22 (0.86,1.71)
Saskatchewan	1.05 (0.84,1.32)	1.26 (0.90,1.75)	0.89 (0.66,1.20)
Depression			
No depressive symptoms	1	1	1
Depressive symptoms	1.79 (1.62,1.97)	1.74 (1.50,2.01)	1.82 (1.61,2.07)
Functional limitations			
No limitations	1	1	1
Some limitations	2.94 (2.51,3.44)	3.34 (2.51,4.44)	2.78 (2.30,3.36)

Note: ^a^Provinces are listed in alphabetic order. Quebec was used as the reference group because it had the lowest prevalence of multimorbidity

## Discussion

This study describes the epidemiology of multimorbidity among middle-aged people based on a national sample of Canadians. Our analysis showed that multimorbidity, defined in this study as having three or more chronic physical health conditions, is a very common occurrence among middle-aged Canadians. The prevalence of multimorbidity was 39.6% in our study population overall, and ranged from 29.7% in the 45–49-year-old age group to 52% in individuals aged 60–64 years. In addition, we found that a substantial proportion of middle-aged Canadians have four or more (24.5%) or five or more chronic physical health conditions (14.2%). Our finding is consistent with the arguments made by several authors that a substantial number of younger people live with multimorbidity. For example, based on a general population sample, Agborsangaya and colleagues [7] reported that 70% of people with multimorbidity were less than 65 years of age. Similarly, Barnett and colleagues [6] reported that the absolute number of people with multimorbidity was higher in those younger than 65 years compared to those older than 65 years of age.

Prevalence estimates of multimorbidity vary widely between studies and comparisons are complicated because definitions of multimorbidity differ (e.g., 2+ vs. 3+ chronic conditions, different number and types of chronic conditions) [43]. Moreover, many studies have focused on primary care samples [43]. As compared to a previous Canadian study that also used a national, general population sample [23] and a 3+ multimorbidity definition, our prevalence estimates are much higher. That study showed that among Canadians aged 20 or older 3.9% had three or more chronic conditions. When examining the prevalence for specific age groups, 4.7% of 50 to 64 year old individuals had three or more chronic conditions. In that study, multimorbidity was defined based on nine chronic conditions. This is contrast to our study, where we included 27 chronic conditions. As has previously been noted, not surprisingly, prevalence rates are higher when more chronic conditions are included [43].

Including a wide range of chronic conditions is useful when focusing on middle-aged individuals as many conditions can significantly impact their lives. In this respect, research shows that the conditions that impact health care utilization differ between younger and older adults [44]. For example, having back problems and migraines was related to increased hospital use among Canadians under 60 years or age, but not among those over 60 years of age [44]. Chronic conditions also have labor force implications among working age people. Rees and Sabia [45] showed, for instance, that having migraines, one of the chronic conditions included in the present study, was associated with reduced labor force participation and lower wages among women.

The association between multimorbidity and lifestyle factors has, to date, received relatively little attention in previous research. In the present study, smoking was not related to multimorbidity. Findings regarding smoking and multimorbidity have been inconsistent in previous research, with one study showing it was negatively related to multimorbidity [31], whereas another study showed no statistically significant relationship [32]. As our measure focused on whether individuals currently smoked, it may be that those with chronic health conditions had stopped smoking. Physical activity was also not related to multimorbidity in the present study, consistent with previous research [31, 34], but contrary to other studies that found statistically significant associations. This discrepancy in findings may be due to the age of study participants; significant relationships between exercise and multimorbidity have been found for older adults, but not younger individuals [30–34].

We further found that alcohol consumption was negatively associated with multimorbidity, unlike previous research that showed no association [30, 32]. People who reported drinking daily or weekly had significantly lower odds of having multimorbidity than those who reported never drinking. This finding is interesting because some studies have shown that moderate alcohol use may have protective effects against some chronic diseases, such as dementia, ischemic heart disease, and stroke [46–48]. In contrast, a recent large meta-analysis showed that alcohol use was a leading risk factor for mortality [49]. A number of explanations may account for the positive association in our study. First, people with high alcohol use would be less likely to participate in a population study. Second, given the relationship between alcohol use and mortality, our sample would consist of relatively healthier individuals. Third, our analyses are cross-sectional in nature, and causality cannot be inferred. It is, therefore, possible that people with multimorbidity stopped drinking. Nevertheless, our finding warrants more research, especially longitudinal analyses to disentangle the temporal relationship between variables. Also, which amount and what kind of alcohol may exert protective effects against multimorbidity would need investigation.

Socio-demographic risk factors for multimorbidity have been studied extensively in previous studies, but some key findings from our study merit attention. First, consistent with previous studies, we found that women had higher odds of multimorbidity than men. Although an association of multimorbidity with female sex has consistently been reported in previous studies [2, 3, 24], an argument has been made that this could be due to the shorter life expectancy of men [3]. Given that selective survival could not be at play in the present study, our finding that the risk of multimorbidity is higher among women than men suggests that this sex difference is a real effect.

Second, like previous studies, we found that low income was strongly associated with multimorbidity [1, 6, 22, 25]. Barnett and colleagues [6], for example, reported that the onset of multimorbidity can occur 10 to 15 years earlier for individual in the lowest socioeconomic group. In the present study, individuals in the lowest income group had almost twice the odds of having multimorbidity compared to those in the highest income group. Education was not related to multimorbidity in the present study, however. This may be because the sample is relatively well educated overall. In the context of a relatively well educated population, income differences may be more important than educational background.

Our study has strengths that are worth mentioning. The sample of our study is a national selected, generalizable sample of the Canadian population. The prevalence and associated determinants we found are therefore generalizable to the overall middle-aged Canadian population. An important consideration is also that we were able to capture 27 chronic conditions while estimating the prevalence of multimorbidity. In contrast, most of the previous studies were based on only a limited number of chronic conditions. Despite these strengths, several limitations must also be acknowledged. First, our results may be subject to recall bias since the measure of multimorbidity was based on self-reports of clinical diagnosis. Second, as this study is mostly cross-sectional in nature, it can merely show associations and causation cannot be inferred. Third, although all the multivariable analyses were controlled for potential covariates, other confounders may exist, such as ethnic background. Finally, our lifestyle measures were broad. For example, the alcohol intake measure did not take into account what people are drinking.

## Conclusions

Given the large number of aging baby boomers, it is important to understand the prevalence and risk factors of multimorbidity to guide clinical care and preventive strategies [50]. In this study, we have described the prevalence of multimorbidity in a national sample of middle-aged Canadians and examined whether lifestyle factors are associated with multimorbidity. The finding that multimorbidity has a high prevalence in this age group suggests the need to focus on prevention, as well as appropriate care and treatment of people with multimorbidity. Additionally, this study identified some areas that require further research, especially the need for longitudinal research on the possible effects of lifestyle on multimorbidity.

## Abbreviations

CATI: Computer Assisted Telephone Interviewing
CCHS: Canadian Community Health Survey
CES-D10: Center for Epidemiologic Studies Short Depression
CHF: Congestive Heart Failure
CI: Confidence Interval
CLSA: Canadian Longitudinal Study on Aging
COPD: Chronic Obstructive Pulmonary Disease
DCS: Data Collection Site
IBD: Inflammatory Bowel Disease
IBS: Irritable Bowel Syndrome
OARS: Older Americans Resources and Services
